# Spatiotemporal crosstalk among mitochondrial dynamics, NLRP3 inflammasome activation, and histone lactylation drives *α*-synuclein pathology in prodromal Parkinson’s disease

**DOI:** 10.3389/fncel.2025.1636185

**Published:** 2025-09-18

**Authors:** Peizhu Lv, Xia Chen, Shiping Liu, Yu Zhang, Yan Bai, Shun Wang, Yulin Wang

**Affiliations:** 1 The Second Clinical Medical College, Heilongjiang University of Traditional Chinese Medicine, Harbin, China; 2 Institute of Acupuncture and Moxibustion, Heilongjiang Academy of Traditional Chinese Medicine, Harbin, China

**Keywords:** Parkinson’s disease, prodromal phase, mitochondrial dynamics, NLRP3 inflammasome, lactylation modification

## Abstract

This article conducts a systematic search of literature in the fields of neuroscience, cell biology, immunometabolism, etc. from 1990 to 2025, with PubMed/WebofScience as the core database. Experimental and clinical studies covering the core mechanisms of the preprophase of PD (mitochondrial imbalance → NLRP3 activation → lactation modification → *α* -SYN pathology) were included, and non-interaction mechanisms and clinical-phase studies were excluded. The pathological interaction network of mitochondrial dynamic imbalance, lysosomes - mitochondrial interaction disorder and neuroinflammation in Parkinson’s disease (PD) was explained. Construct a three-dimensional pathological network of “energy-inflammation-protein homeostasis” to provide a theoretical basis for early intervention. The imbalance of mitochondrial fission/fusion leads to the accumulation of fragmented mitochondria, triggering energy metabolism disorders and oxidative stress; abnormal aggregation of *α*-synuclein (α-syn) disrupts mitochondrial-endoplasmic reticulum membrane (MAM) calcium signaling, upregulates Miro protein to inhibit mitochondrial autophagy clearance, forming a vicious cycle of neuronal damage. Defects in the PINK1/Parkin pathway and LRRK2 mutations interfere with the turnover of mitochondrial fission complexes, causing mtDNA leakage, activating the NLRP3 inflammasome, and driving neuroinflammatory cascades. Additionally, lysosomal dysfunction caused by GBA1 mutations exacerbates mitochondrial quality control defects through Rab7 activity imbalance. Abnormal lactate metabolism may influence inflammasome activity through epigenetic regulation, but its role in PD needs further validation. Based on the above mechanisms, a diagnostic strategy for the prodromal phase integrating dynamic monitoring of mitochondrial fragmentation index, lysosomal function markers, and inflammatory factors is proposed, along with new intervention directions targeting Drp1, NLRP3, and the lysosome-mitochondria interface.

## 1 Introduction

The prodromal phase of Parkinson’s disease (PD) constitutes a preclinical stage exceeding 20 years (Braak stages I–III), characterized by cell-type-specific pathology (e.g., glutamatergic neurons in the olfactory bulb, cholinergic neurons in the dorsal motor nucleus of the vagus) and temporospatial progression (brainstem-to-cortex spread per Braak staging) (Braak et al., 2003; Kulkarni et al., 2022). During early disease (Braak I–II), *α*-synuclein (α-syn) preferentially accumulates in the olfactory bulb (glutamatergic neurons) and dorsal motor nucleus (cholinergic neurons), disrupting N-type calcium channels (Cav2.2) to induce calcium dyshomeostasis and mitochondrial oxidative stress. This manifests clinically as olfactory dysfunction (impaired synaptic transmission) and autonomic disturbances (vagal nucleus dysfunction) (Braak et al., 2003; Dryanovski et al., 2013). By Braak stage III, dopaminergic neurons in the substantia nigra are affected, where microglial activation and TNF-*α* release amplify *β*-band oscillations (15–35 Hz) in the basal ganglia, driving pre-motor symptoms (Brown et al., 2001). Mid-prodromally (Braak II–III), microglia shift from a P2Y12^+^ phagocytic state to a CD16^+^ pro-inflammatory phenotype, propagating *α*-syn via tunneling nanotubes to exacerbate synaptic toxicity (Scheiblich et al., 2021). In late prodromal stages (Braak IV), α-syn impairs astrocytic glutamate transporter GLT-1, inducing excitotoxicity and activating the NF-κB pathway to release pro-inflammatory factors (e.g., IL-6), which synergize with microglia to disrupt the blood–brain barrier (González-Rodríguez et al., 2021).

**Figure 1:**
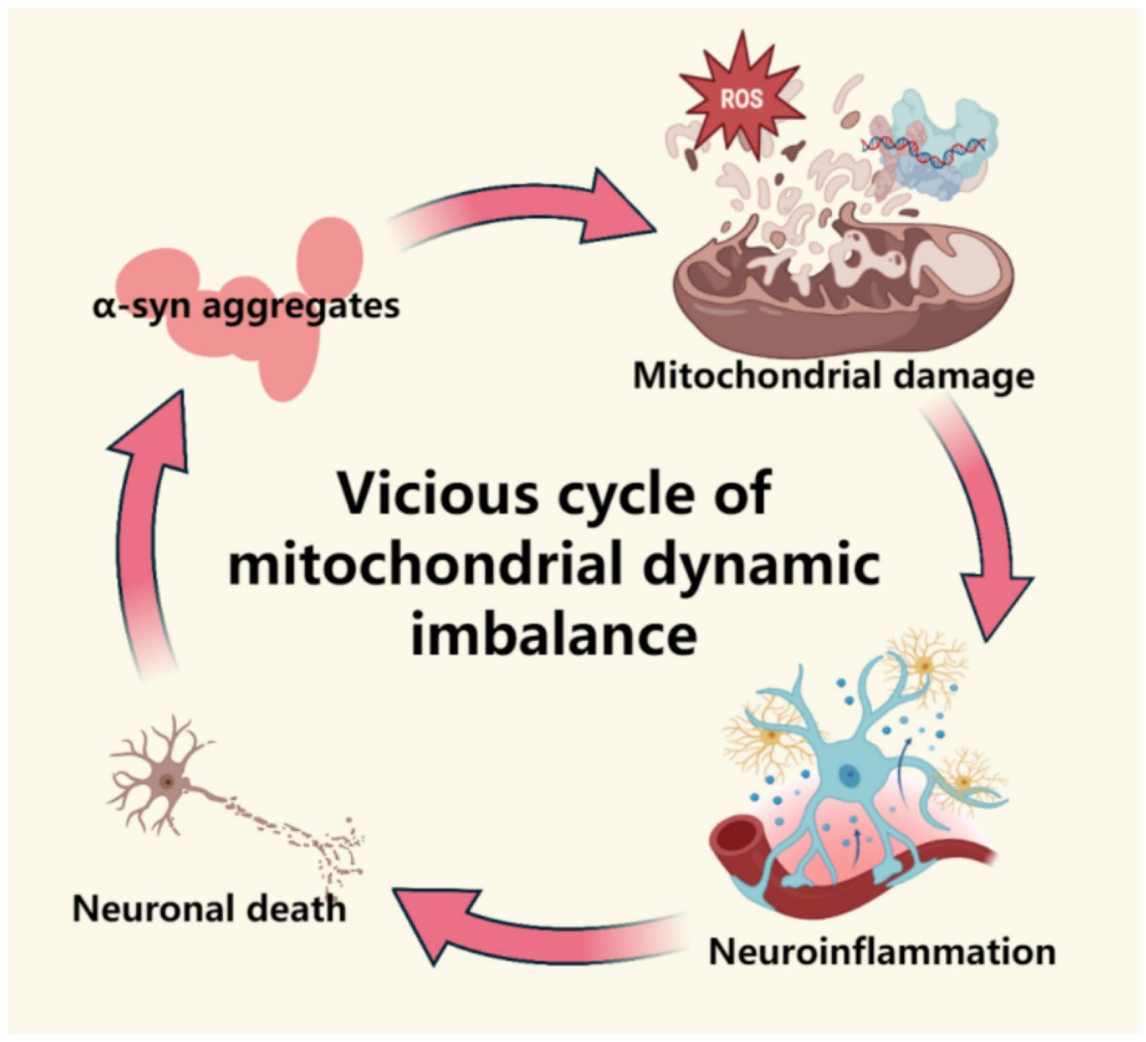
This figure demonstrates the mechanism of mitochondrial dynamic imbalance in the prodromal stage of Parkinson’s disease, including abnormal division/fusion (dysregulation of Drp1 and Mfn1/2), energy metabolism disorders caused by the accumulation of fragmented mitochondria, and the pathological vicious cycle of α -synuclein. This figure was created using the software BioRender.

Despite advances in understanding genetic (e.g., SNCA, LRRK2mutations) and environmental (e.g., MPTP, pesticides) risk factors, motor symptom onset marks irreversible neuronal loss, underscoring the urgency to delineate prodromal mechanisms for early intervention (González-Rodríguez et al., 2021). Previous studies have narrowly focused on isolated pathways—mitochondrial dysfunction, neuroinflammation, or protein aggregation—overlooking their cross-scale network interactions. This work bridges this gap by proposing a unified “Energy-Inflammation-Proteostasis Tripartite Model,” integrating mitochondrial dynamics, lysosomal-mitochondrial metabolic crosstalk, and epigenetic regulation. We elucidate molecular couplings—e.g., Drp1-dependent hyperfission, PINK1/Parkin pathway defects, and NLRP3 inflammasome activation (where LRRK2 enhances inflammation via JNK/NF-κB signaling)—that drive *α*-syn propagation and metabolic decompensation. Our three-tiered framework (molecular interactions, interorganelle metabolic reprogramming, clinical translation) advances novel diagnostic strategies targeting mtDNA damage indices and dynamic IL-1β monitoring, offering transformative avenues for prodromal diagnosis and disease-modifying therapies (see Figure 1).

### 1.1 Mitochondrial dynamics imbalance and its pathological association with PD

The dynamic balance of mitochondrial morphology is maintained by the cooperative action of fusion proteins (Mfn1/2 regulating the outer membrane, OPA1 regulating the inner membrane) and fission proteins (Drp1 and its receptor complexes). Imbalance between the two leads to defects in mitochondrial autophagy and the accumulation of dysfunctional mitochondria (Chan, 2020; Chen et al., 2023; Quintana-Cabrera and Scorrano, 2023). Additionally, mitochondrial transport relies on the microtubule network and associated motor protein complexes, which, in conjunction with dynein, regulate the positioning of mitochondria in neuronal axons, with Mfn2 participating in this process through physical binding to the Miro-Milton complex (Chan, 2020; Gao et al., 2017). In the pathological process of PD, mitochondrial dynamics abnormalities manifest as excessive fission and fusion defects, leading to mitochondrial network fragmentation, energy metabolism disorders, and imbalances in reactive oxygen species (ROS) and calcium homeostasis (Subramaniam and Chesselet, 2013; Rani and Mondal, 2020). A study published in the Journal of Neurochemistry in 1990 first confirmed that the activity of mitochondrial complex I in the substantia nigra of PD patients was significantly reduced compared to healthy controls (decreased by 30%, *p* < 0.05), while the activity of complexes II-III was unaffected (Schapira et al., 1990) Correspondingly, an animal model experiment showed that a single injection of 40 mg/kg MPTP in mice of different ages resulted in age-related declines in complex I function, antioxidant capacity, and increased MAO-B activity, the latter accelerating the conversion of MPTP to toxic metabolite MPP+, suggesting that MPTP-induced oxidative stress and complex I inhibition are potential driving factors of early PD pathology (Ali et al., 1994). To adapt to this functional defect, the density of mitochondria and the expression of complex I/IV proteins in surviving axons increase compensatorily (Reeve et al., 2018). However, the forced enhancement of mitochondrial respiratory chain activity during compensation induces the accumulation of mitochondrial-derived oxidative damage, ultimately triggering or exacerbating neuroinflammatory responses, leading to the collapse of compensatory repair. Pathogenic *α*-syn mutations (e.g., A30P) significantly reduce its localization in MAM by disrupting its interaction with MAM lipid rafts, leading to decreased physical connections between ER and mitochondria (lower Mander coefficient) and impaired calcium signaling mediated by MAM (Guardia-Laguarta et al., 2014; Jiao et al., 2025); this subsequently leads to reduced mitochondrial calcium uptake and energy metabolism disorders (Calì et al., 2012; Devi et al., 2008). Notably, this fragmentation is not driven by the classical fission pathway mediated by DRP1 but is associated with abnormal cleavage of OPA1 protein, suggesting that *α*-syn may indirectly affect morphology by regulating mitochondrial fusion-related proteins (Duvezin-Caubet et al., 2006; Guardia-Laguarta et al., 2014). Furthermore, the reduced presence of mutant *α*-syn in MAM may promote its abnormal accumulation in the mitochondrial membrane, directly interfering with mitochondrial membrane potential and exacerbating oxidative stress by inhibiting complex I activity (Devi et al., 2008), thus forming a vicious cycle of calcium imbalance, energy metabolism disorders, and oxidative damage.

Abnormal accumulation of α-synuclein delays mitochondrial autophagy by upregulating Miro protein levels. Specifically, the clearance rate of damaged mitochondria on the surface of Miro decreases, leading to delayed mitochondrial transport and ultimately resulting in the accumulation of abnormal mitochondria within neurons (Shaltouki et al., 2018). Miro’s abnormal accumulation on the surface of damaged mitochondria causes the motor protein complex (kinesin/Miro/milton) to continuously bind, leading to excessive transport of mitochondria to the distal axon and their retention (Lee and Lu, 2014). Retained mitochondria produce ROS due to electron transport chain dysfunction, and local oxidative stress exacerbates synaptic damage and mtDNA leakage (Verma et al., 2023). Notably, in PD patients, mutations in PINK1 prevent the phosphorylation of Miro at the Ser156 site, leading to the obstruction of Parkin-dependent ubiquitin degradation (Shlevkov et al., 2016; Narendra et al., 2012). The absence of Parkin directly blocks mitochondrial autophagy, resulting in the accumulation of abnormal mitochondria in neuronal cell bodies and axons, ultimately leading to selective death of dopaminergic neurons (Liu et al., 2012; Wang et al., 2011a,b). Accordingly, it can be reasonably inferred that retained damaged mitochondria release DAMPs, such as mtDNA and ROS, activating the NLRP3 inflammasome in microglia and triggering the IL-1β/IL-18 cascade, forming a vicious cycle of “protein abnormal aggregation-mitochondrial damage-neuroinflammation-neuronal death.”

### 1.2 Upstream events related to mitochondrial dynamics imbalance in PD

**Figure 2:**
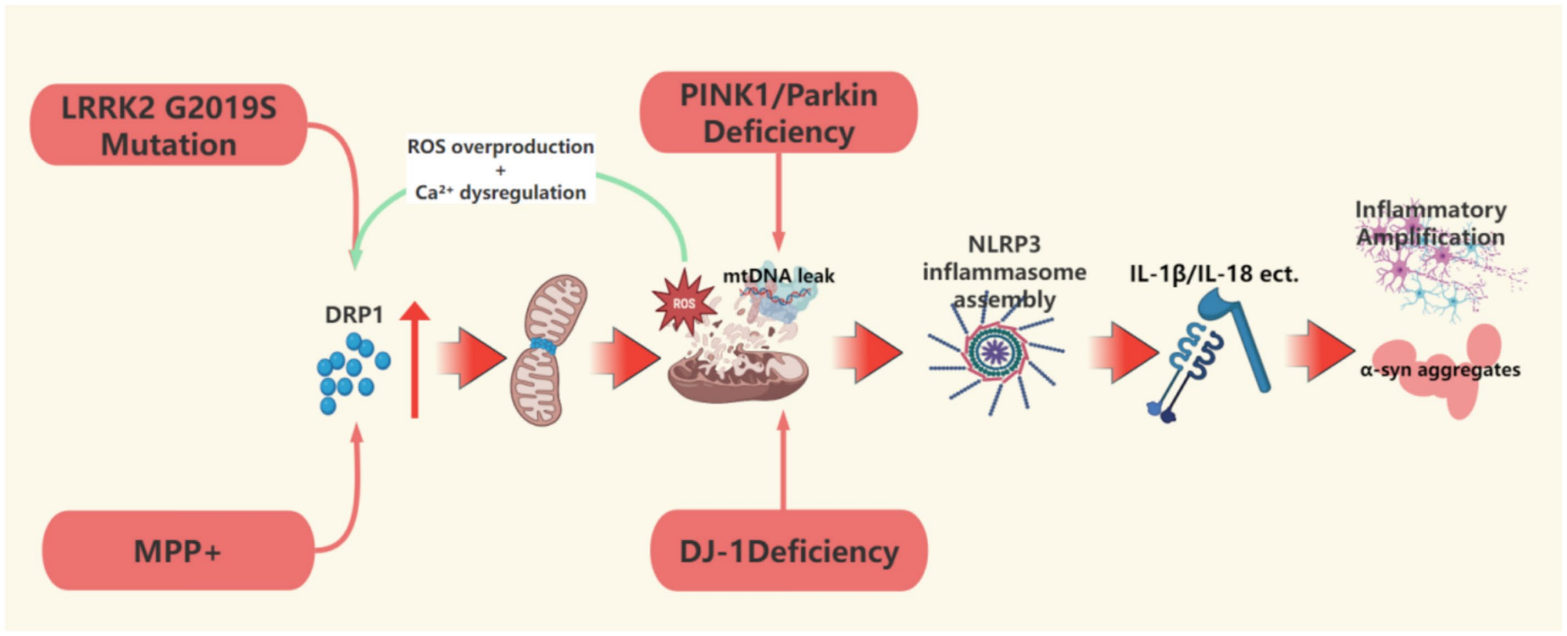
This figure describes the upstream event mechanism of mitochondrial dynamic imbalance, focusing on how DRP1-dependent hyperdivision, defects in the PINK1/Parkin pathway, and LRRK2 mutations induce excessive mitochondrial division, mtDNA leakage, and NLRP3 inflammasome activation, driving the molecular cascade of neuronal damage. This figure was created using the software BioRender.

Studies have shown that Drp1-dependent fission is a key upstream event in the pathology of PD (Zhang D. et al., 2019; Zhang Q. et al., 2019). MPP + induces energy metabolism disorders by inhibiting mitochondrial complex I, activating Drp1-mediated excessive mitochondrial fission, leading to mitochondrial fragmentation (Santos et al., 2015; Chuang et al., 2016). The ROS produced by fragmented mitochondria and abnormal calcium signaling further positively feedback to enhance Drp1 activity, forming a vicious cycle that ultimately leads to the death of dopaminergic neurons. Inhibiting Drp1 can block fragmentation, almost completely rescuing MPP + -induced ROS generation, loss of mitochondrial membrane potential, and cell death (Santos et al., 2015; Yang et al., 2021; Wang et al., 2011a,b). Based on the above mechanisms, targeting the inhibition of Drp1 activity or regulating its mitochondrial translocation may effectively delay the degeneration of dopaminergic neurons in the prodromal phase of Parkinson’s disease by blocking the vicious cycle of mitochondrial fragmentation-ROS-calcium imbalance, providing a new strategy for early neuroprotection (see Figure 2).

For example, mutations in the Parkin and PINK1 genes lead to early-onset Parkinson’s disease, with mechanisms involving defects in the PINK1/Parkin pathway that cause abnormal mitochondrial quality control. When the E3 ubiquitin ligase activity of Parkin is impaired, the abnormal ubiquitination of Mfn1/2 hinders mitochondrial fission and autophagosome formation, resulting in decreased mitochondrial clearance capacity (Gegg et al., 2010). In PINK1 knockout rat models, dopaminergic neurons in the substantia nigra exhibit impaired mitochondrial respiratory function and elevated oxidative stress levels, confirming that mitochondrial homeostasis imbalance is associated with PD pathology (Wang et al., 2023). Defects in the PINK1/Parkin pathway can induce the leakage of mitochondrial DNA (mtDNA) into the cytoplasm, activating the cGAS-STING pathway as a damage-associated molecular pattern (DAMP), which in turn triggers NLRP3 inflammasome assembly and promotes the release of pro-inflammatory factors such as IL-1β and IL-18 (Wang et al., 2023; Mouton-Liger et al., 2018). The absence of Parkin weakens the negative feedback regulation of NF-κB signaling by downregulating the anti-inflammatory protein A20, further amplifying NLRP3 inflammasome activity (Mouton-Liger et al., 2018). Meanwhile, PINK1 deficiency exacerbates the loss of mitochondrial membrane potential and calcium homeostasis imbalance, activating inflammatory responses in microglia and macrophages, leading to a chronic neuroinflammatory microenvironment (Wang et al., 2023). The continuous release of pro-inflammatory factors such as IL-1β and TNF-α synergizes with mitochondrial dysfunction, resulting in oxidative stress, energy metabolism exhaustion, and abnormal aggregation of α-synuclein in dopaminergic neurons, ultimately triggering neuronal apoptosis and degeneration of the nigrostriatal pathway (Wang et al., 2023). The LRRK2 G2019S mutation causes excessive mitochondrial fission by enhancing the phosphorylation of Drp1 at the Thr595 site, accompanied by increased levels of reactive oxygen species (ROS) and mtDNA damage (Su and Qi, 2013). This damage is manifested as a reduction in mtDNA copy number and its cytoplasmic leakage, directly activating the NLRP3 inflammasome (Pena et al., 2024). He et al. (2023) confirmed that the LRRK2 G2019S mutation enhances NLRP3 activation in astrocytes through the NF-κB pathway, promoting the release of IL-1β and TNF-α, a mechanism validated in mouse models and lymphoblasts derived from patients, with LRRK2 kinase activity inhibition significantly reducing inflammasome activity. Zhu et al. (2013) further revealed that LRRK2 G2019S enhances ULK1-dependent mitophagy by activating the JNK signaling pathway, exacerbating mitochondrial clearance abnormalities and neuronal damage. These studies suggest that defects in the PINK1/Parkin pathway and LRRK2 mutations accelerate the pathological process of PD through mitochondrial stress-related inflammatory signaling, and early intervention during the prodromal phase could target the inhibition of NLRP3 or enhance the expression of the anti-inflammatory protein A20 to delay neuronal loss (Wang et al., 2023; Mouton-Liger et al., 2018). Notably, the mtDNA damage caused by the LRRK2 G2019S mutation due to abnormal kinase activity exhibits significant dynamic reversibility. Treatment with EB-42168 or MLi-2 for 2 h to acutely inhibit G2019S LRRK2 kinase activity can rapidly restore mtDNA damage to normal levels, while the damage phenotype reappears within 2 h after the withdrawal of the inhibitor (Pena et al., 2024). This suggests that mitochondrial dysfunction during the prodromal phase may be in a “metabolic imbalance” state rather than structural damage, and this dynamic balance of damage-repair characteristics provides a critical time window for early intervention based on kinase activity monitoring. Dynamic monitoring of mtDNA damage indices and inflammatory factors may construct a combination of prodromal-specific biomarkers, while the combined application of gene editing technology for targeted mtDNA repair and selective kinase inhibitors may achieve precise regulation from molecular mechanisms to clinical phenotypes. Additionally, using induced pluripotent stem cells to construct patient-specific brain organoid models will provide a new platform for optimizing personalized treatment plans.

**Figure 3:**
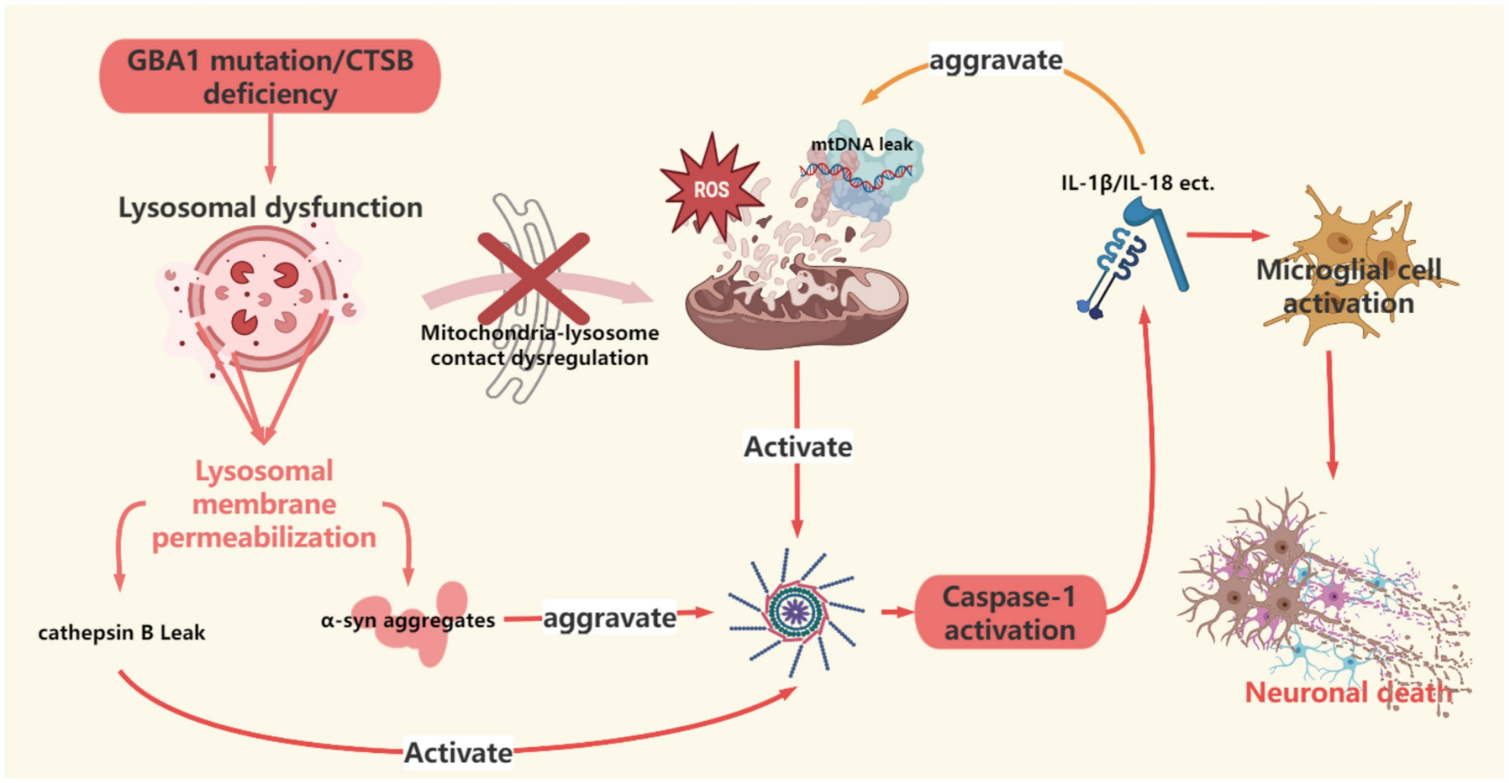
This figure clarifies the pathological interaction mechanism between the mitochondrial-lysosomal interaction disorder and the NLRP3 inflammasome, including lysosomal membrane permeation (CTSB leakage), mtDNA release, and the cascade amplification of inflammatory factors (such as IL-1β), forming a positive feedback loop of α -synuclein diffusion and neuroinflammation. This figure was created using the software BioRender.

Recent studies indicate that DJ-1 is a key downstream mediator of PINK1/parkin-dependent mitophagy (Thomas et al., 2011; McCoy and Cookson, 2011). In fibroblasts and induced dopaminergic neurons from PARK7 mutation patients, the absence of DJ-1 leads to the failure of the autophagy receptor optineurin to be recruited to depolarized mitochondria, directly hindering the clearance of damaged mitochondria and causing the accumulation of mitochondrial fragments (Imberechts and Vandenberghe, 2023). Experiments confirm that the mitochondrial localization of DJ-1 depends on the PINK1/parkin pathway, and artificially targeting DJ-1 to the outer mitochondrial membrane can bypass upstream defects to directly restore optineurin recruitment (Imberechts et al., 2022). These findings indicate that DJ-1 plays a critical role in maintaining mitochondrial quality control, and its deficiency, by blocking downstream steps of autophagy, contributes to the degenerative changes in dopaminergic neurons in conjunction with PINK1/parkin mutations, representing a common pathological mechanism of autosomal recessive PD (see Figure 3).

### 1.3 Pathological interaction between mitochondrial dynamic imbalance and NLRP3 inflammasome

The NLRP3 inflammasome, as a core regulatory component of innate immunity, is closely related to the neuroinflammatory process and loss of dopaminergic neurons in PD. This complex is composed of the NLRP3 receptor protein, the adaptor protein ASC, and the precursor caspase-1 (Lamkanfi and Dixit, 2014), which triggers polymer assembly and activates caspase-1 by sensing danger signals such as intracellular potassium efflux and mitochondrial reactive oxygen species (ROS) bursts (Fu and Wu, 2023; Zhang et al., 2023), subsequently cleaving pro-IL-1β and pro-IL-18 to generate mature inflammatory factors while inducing pyroptosis (Swanson et al., 2019). Co-culture experiments show that NLRP3-activated microglia significantly increase the death of dopaminergic neurons (SH-SY5Y and MN9D cells) (Lee et al., 2019).

In PD patients, mitochondrial ROS serve as the initial signal promoting NLRP3 activation (Mishra et al., 2021), and Parkin can directly regulate NLRP3 stability. The absence of Parkin leads to the accumulation of NLRP3 protein in microglia, enhancing caspase-1 activation and IL-1β secretion, ultimately resulting in the loss of dopaminergic neurons in the substantia nigra (Yan et al., 2023). Specifically, mitochondrial autophagy impairment hinder s the clearance of damaged mitochondria, and the released ROS and mtDNA activate neuroinflammation through dual mechanisms (Lin and Beal, 2006; Yu et al., 2025): on one hand, excessive ROS production due to mitochondrial dysfunction directly promotes NLRP3 inflammasome assembly (Sarkar et al., 2017; Zhou et al., 2011); on the other hand, the released mtDNA from damaged mitochondria synergizes with ROS, further activating the NLRP3 inflammasome by enhancing mitochondrial membrane potential depolarization and lysosomal rupture, driving caspase-1-dependent maturation and secretion of IL-1β (Shao et al., 2021; Javed et al., 2020; Holley and Schroder, 2020). Additionally, recent studies indicate that LPS-induced metabolic reprogramming enhances glycolysis and succinate accumulation in the TCA cycle, increasing mitochondrial membrane potential (Δψm) by reducing F1F0-ATP synthase-dependent proton backflow, while succinate oxidation maintains the pool of reduced CoQ, collectively driving complex I reverse electron transport (RET)-dependent mtROS bursts that regulate NLRP3 inflammasome activation and IL-1β release (Casey et al., 2025). Furthermore, studies have shown that the oxidized sterol metabolite 27-hydroxycholesterol (27-OHC) triggers a cascade of damage by inducing lysosomal membrane permeabilization (LMP): the destruction of lysosomal membrane integrity leads to abnormal leakage of cathepsin B (CTSB), which activates the NLRP3 inflammasome and drives the cleavage of the pyroptosis execution protein GSDMD in a caspase-1-dependent manner, directly causing neuronal inflammatory death (Chen et al., 2019); on the other hand, the abnormal localization of CTSB further exacerbates lysosomal dysfunction, forming a vicious cycle. It is noteworthy that the functional defects of CTSB have a multiple amplification effect—recent studies reveal that CTSB gene knockout or inhibition leads to a triad effect of decreased lysosomal degradation capacity: reduced GCase activity (synergistic effect with GBA1 mutations), autophagic flow blockage, and compensatory increase in lysosomal generation but decreased degradation capacity. This lysosomal collapse state significantly enhances the pathological aggregation of phosphorylated *α*-synuclein (pSyn-S129) induced by α-synuclein preformed fibrils (PFF), directly leading to α-synuclein aggregation, exacerbating the pathological progression of PD, and potentially initiating an irreversible neurodegenerative process during the prodromal phase of PD (Jones-Tabah et al., 2024; Moors et al., 2024).

The underlying mechanisms of this pathological network are closely related to mitochondrial-lysosomal interaction disorders. GBA1 mutations lead to decreased *β*-glucocerebrosidase (GCase) activity, triggering the accumulation of glucosylceramide (GlcCer), which degrades TBC1D15 through the ubiquitin-proteasome pathway, ultimately hindering Rab7-GTP hydrolysis, prolonging mitochondrial-lysosomal (M/L) contact time, and inhibiting Drp1-mediated fission and PINK1-Parkin pathway-dependent autophagy initiation (Kim et al., 2021; Xie et al., 2025), forming a dual defect in mitochondrial quality control. Additionally, Parkin mutations significantly reduce M/L contact frequency by decreasing Rab7 activity (dependent on its E3 ubiquitin ligase function), leading to a deficiency of essential amino acids for mitochondria, such as isoleucine and valine, while causing amino acid accumulation in the lysosomal lumen. This not only impairs mitochondrial oxidative phosphorylation function but also further weakens lysosomal clearance capacity by inhibiting the maturation of lysosomal enzymes such as cathepsin D (Peng et al., 2020). This interaction disorder creates a chain reaction of “quality control failure-metabolic disorder” in a spatiotemporal dimension: GBA1/Parkin mutations lead to abnormal contact time/frequency, while CTSB defects disrupt metabolic terminal clearance capacity, intertwining *α*-synuclein aggregation and the release of inflammatory factors. The IL-1β released after NLRP3 activation exacerbates α-synuclein aggregation and oxidative stress by activating the P38/STAT1 pathway, while IL-6 secreted by neurons enhances the secretion of pro-inflammatory factors from microglia through the JAK2/STAT3 signaling pathway, forming a cascading amplification effect of “inflammatory factors-protein aggregation” (Lee et al., 2023; Chen et al., 2021).

It is noteworthy that the characteristic pathological product of PD—fibrillated *α*-synuclein aggregates—can also act as an endogenous danger signal to activate the NLRP3 inflammasome: studies have confirmed that microglia phagocytose α-synuclein fibrils derived from Lewy bodies, releasing cathepsin B through lysosomal membrane rupture, accompanied by a large generation of ROS, which synergistically activates the NLRP3-caspase-1 axis, driving the maturation and release of IL-1β (Yu et al., 2024; Li G. et al., 2024; Li M. M. et al., 2024; Codolo et al., 2013; Martinon et al., 2002). The released IL-1β further recruits and activates surrounding glial cells, forming a chronic neuroinflammatory microenvironment that exacerbates mitochondrial dysfunction in dopaminergic neurons (Halle et al., 2008). Abnormally aggregated fibrillar α-synuclein drives the inflammatory response through dual mechanisms: on the one hand, by activating Toll-like receptor 2 (TLR2) and TLR3, it synergistically enhances the nuclear factor κB (NF-κB) signaling pathway and upregulates the gene expression of TNF-*α* and IL-1β (Wang et al., 2019; Daniele et al., 2015), and on the other hand, it relies on lysosomal rupture, reactive oxygen species generation, and cathepsin B release to promote the maturation and secretion of IL-1β by triggering the NLRP3 inflammasome, thereby amplifying neuroinflammation (Fan et al., 2020). This forms a positive feedback loop of “*α*-synuclein deposition-inflammation amplification-neuronal death” (Codolo et al., 2013). Genetic evidence further supports this mechanism: αSyn-induced neuroinflammation and motor dysfunction are significantly alleviated in NLRP3 gene knockout mice (Gordon et al., 2018). In summary, the activation mechanism of the NLRP3 inflammasome and its role in PD provide important clues for understanding the pathological mechanisms of this disease, while also laying the foundation for developing new therapeutic strategies. Future research needs to further explore the regulatory mechanisms of the NLRP3 inflammasome and its potential applications in PD treatment.

Based on existing research evidence, during the prodromal phase of PD, the interplay between mitochondrial-lysosomal interaction disorders and neuroinflammation creates a unique diagnostic and intervention window. The ROS and mtDNA release caused by mitochondrial autophagy defects can be detected through the following pathways: elevated mtDNA fragments in cerebrospinal fluid, abnormal mitochondrial membrane potential (Δψm), and activation markers of the NLRP3 inflammasome such as caspase-1 cleavage products and IL-1β levels. Lysosomal function assessment should focus on CTSB activity detection, lysosomal membrane stability markers, and GCase activity evaluation (especially for GBA1 mutation carriers). Notably, the oligomer levels of phosphorylated *α*-synuclein (pSyn-S129) combined with metabolomics analysis may reveal the interaction between “metabolic reprogramming-α-syn pathology, “providing dynamic evidence for risk assessment of the transition from prodromal to clinical stages. Additionally, methods to detect NLRP3 ubiquitination levels in peripheral blood mononuclear cells, such as immunoprecipitation-mass spectrometry combined techniques, can be developed to assess Parkin functional status, providing warning markers for the prodromal phase of PD. It is emphasized that the sensitivity and specificity of this method still need clinical cohort validation, and interference from other E3 ubiquitin ligases must be excluded.

Intervention strategies should be based on the spatiotemporal characteristics of the pathological network: at the metabolic level, drugs that inhibit reverse electron transfer (RET) of complex I block glycolysis-enhanced mtROS bursts (Casey et al., 2025); supplementing essential mitochondrial amino acids such as isoleucine and valine to alleviate metabolic compartmentalization imbalance caused by Parkin mutations. To address lysosomal-mitochondrial interaction disorders, targeting the dynamic balance of Rab7 activity to restore the synergy of mitochondrial fission and autophagy (Jimenez-Orgaz et al., 2018; Liang et al., 2023); CTSB activators or lysosomal membrane stabilizers to restore lysosomal degradation capacity (Prieto Huarcaya et al., 2022), blocking the “seed effect” of *α*-syn pathological aggregation. At the level of inflammation regulation, combining NLRP3 inhibitors with necroptosis pathway blockers simultaneously inhibits IL-1β secretion and neuronal inflammatory death (Tantra et al., 2024; Zhou et al., 2024); microglia-specific JAK2/STAT3 pathway inhibitors cut off IL-6-mediated inflammation spread (Su and Qi, 2013). For genetically susceptible populations, integrating enzyme replacement therapy with personalized metabolic interventions is necessary to break the “mutation-metabolism-inflammation” vicious cycle. The core of this multidimensional intervention system lies in the early identification of the pathological critical point of “mitochondrial autophagy defects-lysosomal collapse-inflammation cascade, “through spatiotemporally specific drugs, such as nanocarriers targeting mitochondrial-lysosomal contact regions, to reshape cellular homeostasis before irreversible neurodegeneration, providing a new paradigm for disease-modifying treatment in the prodromal phase of PD.

### 1.4 The catalytic role of lactylation modification

**Figure 4:**
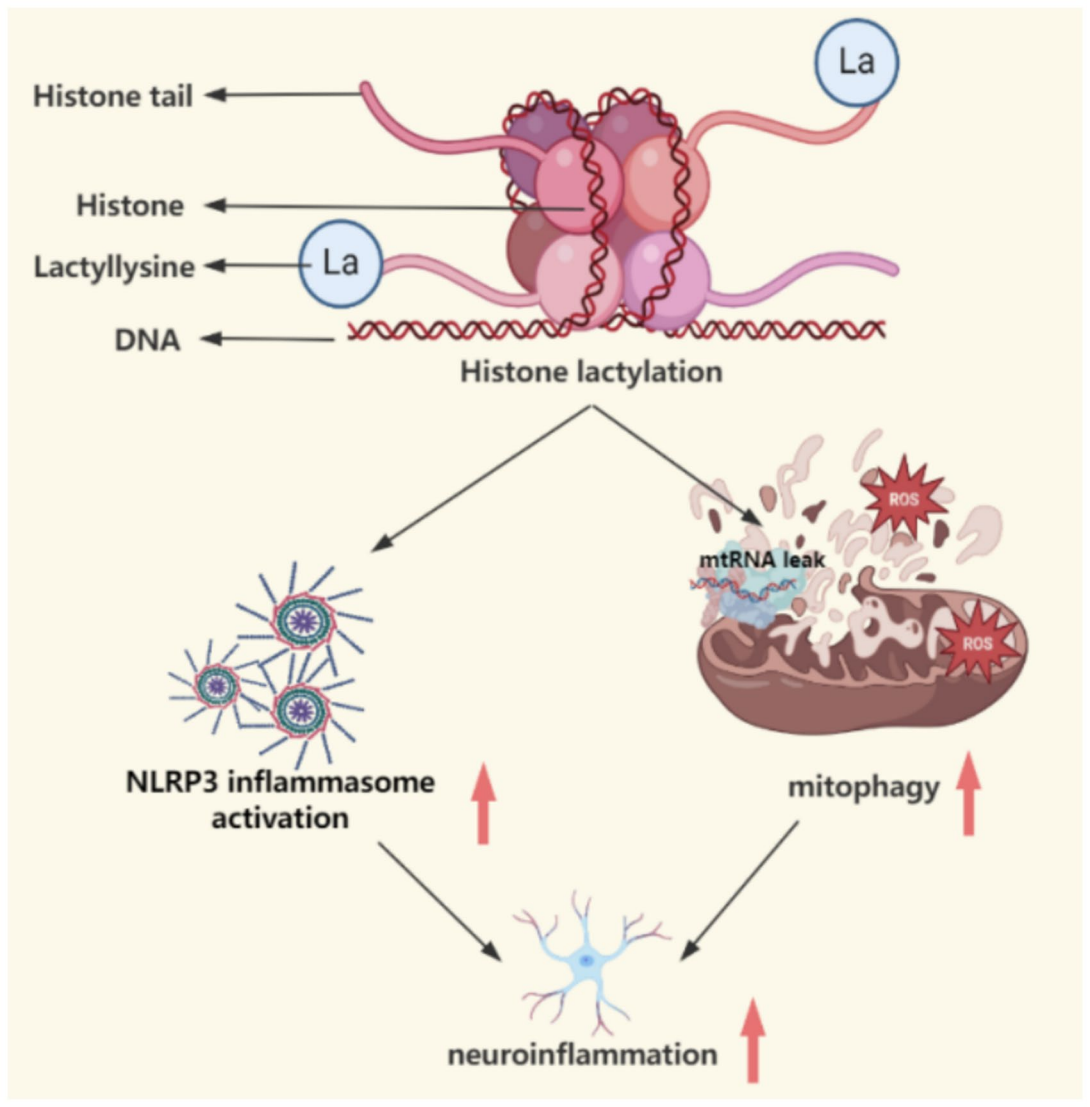
This figure demonstrates the catalytic mechanism of lactic acid modification, which affects mitochondrial function, autophagy deficiency and NLRP3 inflammatory response through metabolic reprogramming (such as enhanced glycolysis) and epigenetic regulation (histone H3K9la modification), connecting the spatiotemporal dynamics of abnormal energy metabolism and neurodegeneration. This figure was created using the software BioRender.

Lactylation modification is a newly discovered epigenetic regulatory mechanism that dynamically regulates protein function through the covalent binding of lactyl groups to lysine residues, becoming a core hub connecting metabolic abnormalities and neurodegenerative diseases (see Figure 4).

Lactylation is involved in physiological processes such as energy metabolism and inflammatory responses. In non-tumor diseases like atherosclerosis and Alzheimer’s disease, lactylation exacerbates pathological damage by regulating inflammation, fibrosis, and cellular senescence (Zhao et al., 2025; Zhang D. et al., 2019; Zhang Q. et al., 2019; Brand et al., 2016). As mentioned earlier, in the pathological process of PD, the abnormal aggregation of α-synuclein has been confirmed to disrupt the integrity of the mitochondria-associated endoplasmic reticulum membrane (MAM), leading to impaired function of mitochondrial complex I and accumulation of reactive oxygen species (ROS) (Guardia-Laguarta et al., 2014). Studies on MPTP-induced Parkinson’s disease models indicate that its toxic metabolite MPP + significantly reduces ATP synthesis efficiency by inhibiting mitochondrial complex I (Ali et al., 1994), prompting cells to activate glycolytic pathways to increase lactate production. This phenomenon shares similar metabolic characteristics with the Warburg effect observed in the tumor microenvironment (Certo et al., 2021). Lactate can inhibit OXPHOS activity through lactylation modification of mitochondrial metabolic enzymes mediated by AARS2, such as PDHA1 K336, forming a negative feedback loop to limit excessive ROS generation (Mao et al., 2024). When this regulatory mechanism is disrupted (e.g., AARS2 deficiency), excessive OXPHOS activity leads to ROS accumulation, potentially triggering mitochondrial oxidative damage and ultimately resulting in the accumulation of dysfunctional mitochondria (Mi et al., 2023). The role of histone lactylation modification in neurological diseases has some experimental support. Research through proteomic analysis has found widespread protein lactylation modifications in mouse brain tissue, and neuronal excitation can significantly increase lactate levels and histone lactylation levels (Hagihara et al., 2021; Kuang et al., 2025; Sun et al., 2025) (Table 1).

**Table 1:** Summary of lactylation biology.

Lactation modification site	Detection methods	Functional mechanisms	Results	References
Mitochondrial complex I proteins	Mitochondrial protein IP-MS	Lactylation inhibits complex I function, aggravating mitochondrial dysfunction and oxidative stress	Parkinson’s disease(PD)	González-Rodríguez et al. (2021)
α-tubulin K40	IP-MS, lactylation-specific antibody WB	HDAC6-catalyzed lactylation enhances microtubule dynamics; alters axonal stability balance, enhances plasticity; impairs dopaminergic neuronal transport function	Neurodegenerative diseases (PD/AD, etc.)	Sun et al. (2024) and González-Rodríguez et al. (2021)
H4K12	CUT&Tag + qChIP; Immunofluorescence staining; Western Blotting	Activates the “glycolysis-H4K12la-PKM2” positive feedback loop, exacerbating microglial dysfunction	Alzheimer’s disease (AD)	Pan et al. (2022)
NLRP3-K245	Lactylation antibody + co-precipitation	LDHA promotes cardiomyocyte pyroptosis	Myocardial ischemia–reperfusion injury	Fang et al. (2024)
Histone H1	Immunohistochemistry (IHC)	Increased neuronal excitability drives Kla in the prefrontal cortex, associated with anxiety-like behavior	Social defeat stress (anxiety model)	Hagihara et al. (2021)
H4K5	ChIP-qPCR	Macrophage phenotypic transition disorder, increased release of pro-inflammatory factors (TNF-α, IL-6), aggravates vascular inflammation	Atherosclerosis (AS)	Yang H. et al. (2022) and Yang K. et al. (2022)
SLC25A4-K245, SLC25A5-K96	Lactylation antibody + co-precipitation	Interfere with the calcium signaling pathway, induce mitochondrial Ca^2+^ overload and neuronal apoptosis	Cerebral ischemia–reperfusion injury (CIRI)	Yao et al. (2023)
HMGB1	ELISA, cellular confocal imaging	Disrupts vascular endothelial barrier	Sepsis	Yang H. et al. (2022) and Yang K. et al. (2022)
APOC2-K70	Immunoprecipitation-mass spectrometry (IP-MS)	Induces tumor metastasis and resistance to immunotherapy	Non-small cell lung cancer (NSCLC)	Chen B. et al. (2024); Chen J. et al. (2024)
H3K18	ChIP-seq、WB	Activates POM121/MYC/PD-L1 pathway to promote immune escape		Zhang et al. (2024)
β-catenin	Immunofluorescence, Co-IP	Activates Wnt signaling pathway to promote proliferation	Colorectal cancer (CRC)	Miao et al. (2023)
NSUN2-K356	Lactylation antibody + MS validation	Promotes tumor progression		Chen B. et al. (2024); Chen J. et al. (2024)
H3K9, H3K56	Histone modification profile analysis	Upregulates ESM1 to enhance malignant phenotype	Hepatocellular carcinoma (HCC)	Zhao et al. (2024)
H3K18	CUT&Tag Anti-H3K18la antibody WB	Activate neuroendocrine genes such as Mycn and Ascl1	Neuroendocrine prostate cancer (NEPC)	Wang et al. (2024)
XRCC1-K247	Anti-XRCC1 lac-K247 antibody Immunoprecipitation mass spectrometry (IP-MS)	Induces therapy resistance	Glioblastoma (GBM)	Li G. et al. (2024); Li M. M. et al. (2024)

In summary, the abnormal accumulation of lactate may exacerbate neuroinflammation through a tripartite attack:

1. By remodeling the promoter regions of glycolysis-related genes through histone lactylation modification, a positive feedback loop is formed involving glycolytic hyperactivity, histone lactylation modification, and transcription of glycolytic genes. Pan et al. (2022) found that in an Alzheimer’s disease model, lactate accumulation due to glycolytic hyperactivity in microglia induces histone H4K12 lactylation modification, activating the transcription of glycolytic genes such as PKM2, further strengthening glycolytic metabolism and forming a self-sustaining vicious cycle, impairing phagocytic function and promoting pro-inflammatory phenotypes, ultimately leading to microglial dysfunction and exacerbated neuroinflammation. Although there is currently a lack of direct research data targeting PD, if lactate levels in the brain increase due to mitochondrial dysfunction or metabolic abnormalities, it may similarly lead to impaired phagocytic function in microglia, enhancing the pro-inflammatory phenotype and resulting in the release of pro-inflammatory factors such as TNF-*α* and IL-1β, exacerbating dopaminergic neuronal damage.
2. Inhibition of PINK1/Parkin pathway-mediated mitophagy leads to the accumulation of damaged mitochondria and the release of ROS, mtDNA, and other DAMPs. Some studies have found that lactylation modification regulates Parkin-mediated mitochondrial quality control by targeting mitochondrial metabolic enzyme PCK2, where lactate activates KAT8 to catalyze lactylation modification at the Lys100 site of PCK2, significantly enhancing its kinase activity. Activated PCK2 competitively inhibits the ubiquitination degradation of the rate-limiting enzyme (OXSM) for mitochondrial fatty acid synthesis by Parkin, leading to increased stability of OXSM protein, which drives abnormal enhancement of mitochondrial fatty acid synthesis and oxidative phosphorylation. This metabolic reprogramming process triggers mitochondrial membrane potential depolarization and ROS overload (Yuan et al., 2025). Based on the above mechanisms, it can be reasonably speculated that in PD, abnormal lactate metabolism may inhibit PINK1/Parkin pathway-mediated mitophagy through similar pathways: lactylation modification may target interacting proteins of PINK1 or Parkin, obstructing the ubiquitination marking and autophagosome encapsulation of damaged mitochondria; defects in mitophagy lead to the accumulation of depolarized mitochondria, leaking large amounts of ROS through the electron transport chain, and releasing damage-associated molecular patterns (DAMPs) such as mtDNA and ATP, activating the NLRP3 inflammasome in microglia; additionally, mitochondrial DAMPs may upregulate the expression of pro-inflammatory factors (such as IL-1β and IL-18) through epigenetic regulation (e.g., histone H3K18la) in conjunction with lactylation modification, synergistically amplifying the neuroinflammatory response. These mechanisms may be related to the pathological spread of *α*-synuclein and the loss of dopaminergic neurons during the prodromal phase of PD, but need to be validated in PD models and the role of specific lactylation targets.
3. Lactylation modification promotes the activation of the NLRP3 inflammasome and the expression of inflammatory factors through epigenetic regulatory mechanisms (You et al., 2024; Wang et al., 2025; Fang et al., 2024). Recent research by Ge et al. directly confirmed through chromatin immunoprecipitation (ChIP) experiments that the enrichment of H3K9la at the NLRP3/ASC promoter is positively correlated with gene expression, revealing that lactate produced by macrophages under LPS stimulation enhances the accessibility of the NLRP3 and ASC promoters through H3K9la modification, promoting inflammasome assembly and the maturation and release of IL-1β, forming a pro-inflammatory microenvironment (Ge et al., 2025). This provides direct evidence for the epigenetic regulatory role of lactylation modification. Accordingly, it can be reasonably speculated that during the course of PD, a similar lactylation-NLRP3 regulatory axis may exacerbate neuroinflammation. The characteristic aggregation of *α*-synuclein in PD can activate the NLRP3 inflammasome in microglia, while the abnormal increase in lactate levels in the brain may enhance the inflammatory response of microglia by promoting H3K9la modification. This mechanism may explain the elevated levels of IL-1β in the cerebrospinal fluid of PD patients and provide a theoretical basis for neuroprotective strategies targeting lactylation modification. Future research needs to verify the spatiotemporal association between lactate metabolism and NLRP3 activation in the microenvironment of midbrain dopaminergic neurons, as well as the regulatory effects of lactate dehydrogenase inhibitors on neuroinflammation.

Lactylation is a key link between metabolism and epigenetic regulation. Some studies have found that lactyl-CoA is a donor for lactylation, and its modification is catalyzed by CBP (CREBBP) or p300 (EP300). CBP/p300 acts as the writer enzyme for lactylation modification, catalyzing the lactylation of histone lysines through its acetyltransferase structural domain (Zhang D. et al., 2019; Zhang Q. et al., 2019; Dai et al., 2022). Histone deacetylases HDAC1-3 have the activity to remove H3K18la and act as “eraser enzymes” for lactylation, inhibiting the transcriptional activity of related genes by hydrolyzing lactylation modifications (Dai et al., 2022). Therefore, targeting the regulation of the activity of writer or eraser enzymes may balance the levels of lactylation in the brain, potentially providing a direction for neuroprotection during the prodromal phase of PD, but further experimental validation is needed. The discovery of lactylation modification provides a new perspective for PD treatment: as a triple regulatory node of metabolism, epigenetics, and inflammation, it can explain the co-occurrence of energy metabolism abnormalities and neuroinflammation in PD, and lay a theoretical foundation for developing multidimensional intervention strategies targeting both the NLRP3 inflammasome and mitophagy. Future studies should precisely analyze the spatiotemporal dynamics of lactylation modification and its interaction mechanisms with *α*-synuclein pathology in PD models using single-cell metabolic imaging combined with CUT&Tag technology.

## 2 Discussion

Mitochondrial dynamic imbalance is a core hub in the pathological process of PD, where network fragmentation, energy metabolism disorders, and calcium homeostasis imbalance together constitute early driving factors for neurodegeneration (Celardo et al., 2014; Rocha et al., 2018). The abnormal aggregation of α-synuclein not only exacerbates the energy metabolism crisis by disrupting calcium signaling in mitochondria-associated membranes (MAM) (Mao et al., 2022; Sironi et al., 2020) but also hinders the autophagic clearance of damaged mitochondria by upregulating Miro protein levels (Shaltouki et al., 2018), ultimately leading to a vicious cycle of abnormal mitochondrial accumulation and oxidative damage within neurons. In this process, although the compensatory increase in mitochondrial density temporarily maintains energy supply, it accelerates oxidative stress and neuroinflammation due to the overload of respiratory chain activity, suggesting that early diagnosis should focus on the dynamic monitoring of mitochondrial network morphology and the precise definition of metabolic compensation thresholds. For example, combining the mitochondrial fragmentation index in the substantia nigra with cerebrospinal fluid mitochondrial complex I activity detection can identify the critical point of transition from the compensatory phase to the decompensatory phase; quantitative analysis of MAM connection proteins may reveal early associations between *α*-syn pathology and mitochondrial calcium imbalance, providing spatiotemporal specific targets for intervention strategies.

In-depth exploration of upstream events of mitochondrial dynamic imbalance reveals that excessive activation of Drp1-dependent fission is not only a pathological core induced by neurotoxins like MPTP but is also closely related to the collapse of mitochondrial quality control networks caused by gene mutations such as VPS35 and PINK1/Parkin. The lysosomal degradation barrier mediated by mitochondrial-derived vesicles (MDVs) leads to abnormal turnover of Drp1, causing excessive assembly of fission complexes and exacerbating mitochondrial fragmentation; mtDNA leakage caused by defects in the PINK1/Parkin pathway promotes NLRP3 inflammasome assembly through the activation of the cGAS-STING pathway, tightly coupling mitochondrial damage with neuroinflammation. These findings suggest that interventions during the preclinical phase need to balance precise molecular-level regulation with systemic-level network repair: on one hand, in-depth studies on the locking of Drp1 conformations or the regulation of Rab7 activity to restore the spatiotemporal coordination of mitochondrial fission-autophagy; on the other hand, targeting the protection of mtDNA integrity or inhibiting excessive activation of the NLRP3 inflammasome may block the cascade reaction of “mitochondrial damage-inflammation amplification” (Li et al., 2020). Notably, DJ-1, as a downstream hub of the PINK1/Parkin pathway, may provide a common therapeutic window across mutation types for autosomal recessive PD through functional replacement strategies, such as mitochondrial-targeted optineurin recruitment-enhancing peptides.

The interaction barrier between mitochondria and lysosomes, along with the pathological interaction of the NLRP3 inflammasome, further expands the intervention dimensions during the preclinical phase of PD. The leakage of CTSB caused by lysosomal membrane permeabilization (LMP) not only directly activates NLRP3 (Meng et al., 2023), but also accelerates pathological protein aggregation by inhibiting *α*-synuclein clearance (Bellomo et al., 2020). In this context, spatiotemporally specific intervention strategies, such as targeting lysosomal membrane stability (e.g., TRPML1 activators) combined with mTORC1 inhibitors, may restore lysosomal degradation function and inhibit oxidative stress driven by the glycolysis-mitochondrial ROS axis (Bonam et al., 2019). Furthermore, the mechanism by which α-syn aggregates activate microglia through the TLR2/NLRP3 dual pathway suggests that immune modulation targeting pathological protein conformations, such as neutralizing antibodies against α-syn oligomers, in combination with inflammatory pathway blockade, may effectively interrupt the self-reinforcing loop of “protein aggregation-inflammation diffusion” during the preclinical phase.

The interaction barrier between mitochondria and lysosomes, along with the pathological interaction of the NLRP3 inflammasome, constitutes a core driving network for the progression of Parkinson’s disease (Sarkar et al., 2017; Abdelaziz, 2025). Genetic defects disrupt mitochondrial-lysosomal contact dynamics, bi-directionally disturbing quality control mechanisms: on one hand, the inhibition of mitochondrial fission protein Drp1 function leads to the accumulation of fragmented mitochondria, while impaired autophagic flow promotes mtDNA leakage and ROS bursts; on the other hand, lysosomal enzyme maturation defects weaken the clearance capacity of α-synuclein aggregates, creating a “seed effect” of abnormal protein deposition. This dual collapse of metabolism and degradation activates the NLRP3 inflammasome through spatial coupling mechanisms: leaked mtDNA triggers inflammasome assembly via the cGAS-STING pathway, while cathepsin B released from lysosomal membrane permeabilization directly cleaves NLRP3, and α-synuclein fibers drive IL-1β maturation through the TLR2/NLRP3 dual signaling axis, ultimately forming a cascade amplification loop of “mitochondrial damage-lysosomal collapse-inflammation storm.” Targeting this pathological network, early diagnosis should establish a multi-dimensional biomarker system: integrating dynamic monitoring of cerebrospinal fluid mtDNA fragment quantification, lysosomal membrane stability indicators, and NLRP3 activation markers, along with α-synuclein oligomer detection and metabolomic feature analysis, can accurately identify the critical point of “compensation-decompensation” transition. Intervention strategies should focus on spatiotemporally specific regulation, restoring mitochondrial-lysosomal contact rhythms, and rationally developing and utilizing targeted Drp1 inhibitors and CTSB activators, while simultaneously inhibiting reverse electron transfer in complex I to reduce mtROS production, effectively restoring the interaction mechanisms between mitochondria and lysosomes and preventing further inflammation spread.

The discovery of lactylation modifications injects a triple regulatory perspective of metabolism-epigenetics-inflammation into the pathological mechanism of PD. The accumulation of lactate caused by mitochondrial dysfunction not only inhibits the transcription of autophagy genes through histone lactylation modifications, leading to the collapse of quality control networks, but also enhances the chromatin accessibility of the NLRP3 promoter, amplifying neuroinflammation. Targeting this mechanism, early diagnosis during the preclinical phase of PD can develop molecular imaging technologies based on dynamic lactate metabolism combined with cerebrospinal fluid lactylation modification marker detection, while integrating assessments of mitochondrial autophagic flow and NLRP3 activation indicators to construct a multi-dimensional early warning system. Intervention strategies should focus on the synergistic regulation of metabolism and epigenetics: designing blood–brain barrier penetrating nanocarriers loaded with LDHA inhibitors and KAT8 antagonists to target and block the cascade effects of lactylation modifications (Cheng et al., 2024); developing spatiotemporally specific gene editing tools to selectively clear lactylation modifications at pro-inflammatory gene loci; and combining targeting mitochondrial homeostasis with the use of NLRP3 inhibitors to break the vicious cycle of “metabolic compensation-inflammation amplification”(Ren et al., 2024; Zhu et al., 2025). These intervention measures need to be validated for their neuroprotective effects in organoid models and gene-edited animals, ultimately achieving a transition from “pathological blockade” to “homeostatic remodeling, “providing a new paradigm for precision medicine in the preclinical phase of PD.

In summary, the pathological network during the preclinical phase of PD exhibits characteristics of multi-level, cross-scale interactions, from mitochondrial fragmentation to epigenetic reprogramming of lactylation modifications, from lysosomal collapse to NLRP3 inflammation storms. Each mechanistic node is not isolated but forms a tightly coupled vicious cycle through energy metabolism imbalance and neuroinflammation diffusion. Early diagnosis needs to break through the limitations of single biomarkers and establish a multi-dimensional assessment system integrating morphological compensation markers, metabolic-epigenetic correlation profiles, and interface interaction dynamics parameters; while intervention strategies should focus on the “fragile nodes” of the pathological network, achieving the remodeling of quality control networks and reversal of inflammatory microenvironments through spatiotemporally specific drugs and gene-metabolism combined regulation within the compensation threshold. Future research needs to validate the synergistic effects of these strategies in PD preclinical animal models and explore the dynamic associations between cerebrospinal fluid exosomal markers and imaging genomics, thereby opening up new paradigms for pre-diagnosis of motor symptoms and disease-modifying therapies.
